# Evaluating the Efficacy of Comprehensive Nursing Care in Improving the Well-Being of Patients With Colon Cancer Who Developed Postoperative Sleep Disorders

**DOI:** 10.62641/aep.2156

**Published:** 2026-10-15

**Authors:** Na Ou, Yajing Xu

**Affiliations:** ^1^Department of Gastrointestinal Surgery, The Second People's Hospital of Changzhou, The Third Affiliated Hospital of Nanjing Medical University, 213000 Changzhou, Jiangsu, China

**Keywords:** colonic neoplasm, postoperative care, anxiety, depression, sleep disorder

## Abstract

**Background:**

This study aimed to investigate the effect of comprehensive nursing care on the psychological state and related indicators of patients with postoperative sleep disorders after colon cancer surgery.

**Methods:**

We retrospectively selected 121 patients with sleep disorders who underwent colon cancer resection in our hospital between January 2021 and December 2023. After 1:1 propensity score matching (PSM), 52 patients were included in the routine nursing group and 52 in the comprehensive nursing group. Comprehensive nursing care, as documented in medical records, included psychological nursing, pain management, rehabilitation guidance and social support care.

**Results:**

Data extracted from medical records and follow-up files showed that the comprehensive nursing group had greater reduction in Self-Rating Anxiety Scale (SAS) and Self-Rating Depression Scale (SDS) scores after surgery than the routine nursing group (*p* < 0.05); the comprehensive nursing group also had shorter first anal exhaust time, first defecation time and hospitalization duration than the routine nursing group (*p* < 0.05). The complication incidence rate in the comprehensive nursing group was lower than the routine nursing group, but the difference was not statistically significant (*p* = 0.080). Improvements in all domains of the Short-Form 36 (SF-36) Health Survey in the comprehensive nursing group were greater than the routine nursing group (*p* < 0.05). The total score and several component scores of the Pittsburgh Sleep Quality Index decreased more significantly in the comprehensive nursing group (*p* < 0.05).

**Conclusions:**

Comprehensive nursing care is a significant correlated between improved psychological state, rehabilitation-related indicators, quality of life and sleep quality in patients with postoperative sleep disorders after colon cancer surgery.

## Introduction

Colon cancer is one of the common malignancies worldwide, with an increasing incidence that seriously threaten human health and quality of life [1, 2, 3]. Surgery is an important treatment modality for colon cancer, but postoperative patients often face a range of physiological and psychological challenges [4, 5, 6].

Physiologically, surgical trauma can lead to pain, gastrointestinal dysfunction and malnutrition [7, 8, 9]. Psychologically, the emotional state of patients after colon cancer surgery is complex and fragile [10, 11]. On the one hand, fear of cancer itself and worry about postoperative recurrence hang over patients like a cloud. During rehabilitation, patients remain highly sensitive to minor bodily changes; every new discomfort may trigger panic about cancer progression. On the other hand, surgery may alter body image. For example, stoma surgery can cause a major psychological impact on patients, leading to feelings of inferiority and shame, affecting normal social life and psychological balance [12, 13].

In current medical practice, increasing evidence shows that patients’ psychological state is closely related to postoperative rehabilitation outcomes [14, 15]. A negative psychological state may delay recovery, reduce quality of life, and even affect long-term survival rate. For example, chronic anxiety and depression may suppress immune function, increase infection risk, and negatively affect adherence to subsequent treatments such as chemotherapy [16]. Therefore, addressing the psychological state of patients after colon cancer surgery and implementing effective nursing care measures has become an important component of clinical nursing.

However, traditional nursing models often focus on physiological care, with relatively insufficient attention to psychological nursing. Although some nursing measures can alleviate psychological problems to a certain extent, they lack systematic and comprehensive approaches. The innovations of this study are reflected in three aspects: firstly, it constructs a multidimensional integrated care system covering psychological nursing, pain management, rehabilitation guidance and social support, breaking the limitations of single-dimension care; secondly, it introduces individualized stratification in pain management, distinguishing the independent effects of nonpharmacological treatments and their synergistic effects with pharmacological treatments, and clarifies the care cycle to ensure the standardization; thirdly, it combines short-term postoperative indicators (e.g., gastrointestinal function recovery, hospitalization duration) with long-term outcomes (e.g., 3-month quality of life, sleep quality) to form a complete evaluation system. Therefore, investigating the effect of comprehensive nursing care on the psychological state of patients after colon cancer surgery is of great practical significance, aiming to explore a more effective nursing model, comprehensively improve patients’ psychological status, and promote postoperative rehabilitation.

## Materials and Methods

### General Information

Between January 2021 and December 2023, we retrospectively identified 121 patients who underwent colon cancer resection in The Second People’s Hospital of Changzhou, the Third Affiliated Hospital of Nanjing Medical University, from the hospital’s electronic medical record system. The diagnosis of sleep disorders for all included patients was jointly made by psychiatrists or sleep medicine specialists in our hospital in accordance with the Diagnostic and Statistical Manual of Mental Disorders, 5th Edition (DSM-5) [17]. Additionally, the Pittsburgh Sleep Quality Index (PSQI) was used for auxiliary quantitative evaluation, with a PSQI score >7 as the quantitative reference for sleep disorder severity [18].

After 1:1 nearest-neighbour propensity score matching (PSM) (calliper width = 0.1, without replacement), 52 patients were included in the routine nursing group and 52 in the comprehensive nursing group. Covariates for PSM included age, sex, body mass Index, education level, smoking/drinking status, tumour location, tumour node metastasis stage [19], and surgical method. Covariate balance was assessed using standardized mean difference (standardized mean difference (SMD) < 0.1 indicates good balance). The sample size was determined based on the total number of patients who met the inclusion and exclusion criteria during the study period, and statistical analysis verified baseline comparability between the two groups, ensuring the scientific validity of the study sample.

The inclusion criteria were as follows: (1) pathologically confirmed colon cancer, underwent surgical resection, and diagnosed with sleep disorders by psychiatrists/sleep medicine specialists in accordance with DSM-5 criteria with a PSQI score >7 [18]; (2) aged ≥18 years; (3) expected postoperative survival >3 months; and (4) patient and family members provided informed consent and signed the relevant consent forms.

The exclusion criteria were as follows: (1) comorbid severe organic diseases (e.g., severe cardiovascular and cerebrovascular diseases, liver and kidney failure); (2) pre-existing mental diseases or cognitive impairment; (3) severe postoperative complications requiring prolonged hospitalization, defined as Clavien–Dindo grade III or above according to the Clavien–Dindo classification of postoperative complications; (4) unable to cooperate with questionnaire completion or nursing care.

### Methods

#### Nursing Plan for the Routine Nursing Group

Routine nursing care was adopted, and the nursing duration was consistent with hospitalization duration (from postoperative day 1 to discharge), including the following contents:

(1) Condition monitoring: Closely observe postoperative vital signs (e.g., body temperature, blood pressure, heart rate, and respiratory), record regularly and report abnormalities to the doctor in a timely manner.

(2) Wound care: Keep the surgical incision clean and dry, change dressings regularly under aseptic technique, observe for bleeding, exudation, redness, swelling and healing, and prevent infection.

(3) Tube care: Properly secure drainage tubes such as the gastric tube and urinary catheters, keep them unobstructed, prevent twisting, compression and detachment, accurately record the amount, colour and nature of the drainage fluid, and remove the drainage tubes promptly as ordered.

(4) Dietary guidance: Based on gastrointestinal function recovery, gradually transition from fasting to liquid diet, semi-liquid diet, soft diet and then normal diet. Guide patients to choose low-fat, high-protein, high-vitamin, and easily digestible foods, avoid irritating foods, ensure nutritional intake, and promote physical recovery.

#### Nursing Plan for the Comprehensive Nursing Group

According to nursing records, the comprehensive nursing group received comprehensive nursing care, implemented from postoperative day 1 to discharge (hospitalization phase). Additionally, medical records and follow-up files indicated that patients received 1 follow-up visit per month for 3 consecutive months after discharge (post-discharge phase) as part of the comprehensive nursing care, which was provided in addition to routine nursing care.

Design philosophy and theoretical basis: The comprehensive nursing care is based on the “biological–psychological–social” medical model and holistic nursing theory. It integrates multiple care dimensions to address the physiological, psychological, and social needs of patients with colon cancer and postoperative sleep disorders, aiming to comprehensive improve patient outcomes. The total care duration is the hospitalization period plus 3 months of post-discharge follow-up.

(1) Psychological nursing

Establish a professional psychological nursing team, including nurses with intermediate or higher professional titles and psychological counsellors holding national second-level psychological counsellor certificates and more than 5 years of clinical psychological care experience. Conduct in-depth communication with patients and their families in the early postoperative period, assess patients’ psychological state, and understand their knowledge of the disease and surgery, psychological concern.

Provided personalized psychological counselling: Each session lasts 30–45 minutes, conducted 2–3 times during hospitalization (once every 3–4 days) and once a month during post-discharge follow-up. Based on psychological assessment results, for emotions such as fear and anxiety, use simple and easy-to-understand language to explain colon cancer knowledge, surgical treatment outcomes, and postoperative rehabilitation precautions, correct misconceptions about the disease, and enhance patients’ confidence in overcoming the disease.

Encourage patients to express their inner feelings, listen patiently to their concerns, provide emotional support, and help patients establish a positive attitude. At the same time, share successful treatment cases, guide patients to communicate with each other, and share rehabilitation experiences to relieve psychological pressure.

(2) Pain management

Establish a pain assessment system using the Visual Analogue Scale (VAS) [20] to assess patients’ postoperative pain level, and adjust the pain management plan promptly based on the assessment results.

Implement multimodal analgesia: Based on our clinical experience, for patients with a VAS score of 1–3, when pain relief is needed, administer oral analgesics as prescribed; for patients with a VAS score of 4–6, administer an appropriate amount of oral analgesics as prescribed; for patients with a VAS score of 7–10, promptly report to the doctor and use strong analgesics (such as opioids) or patient-controlled analgesia pumps as prescribed. Closely observe adverse drug reactions during the analgesia to ensure safe and effective pain management.

Strengthen pain education: Explain the causes of pain, the importance of pain control and how to use analgesic drugs correctly to patients and their families, improving adherence to pain management.

(3) Rehabilitation guidance

Early postoperative activity guidance: Developed by professional rehabilitation nurses, implemented from postoperative day 1until discharge. Formulate personalized activity plans based on patients’ physical condition. Encourage patients to turn over and move their limbs in bed on the first postoperative day; assist patients to sit up and move around the bed on the second postoperative day; gradually increase activity, such as walking in the ward, to promote gastrointestinal peristalsis recovery and prevent pulmonary complications and lower extremity venous thrombosis.

Rehabilitation training: Conducted 3 times a week during hospitalization and once a week during post-discharge follow-up, 30 minutes per session. Based on the patient’s recovery, guide patients in rehabilitation training such as pelvic floor muscle training and abdominal muscle exercise to enhance muscle strength and improve physical function. For example, guide patients to perform anal contraction exercises, contract the anus for 3–5 seconds each time, then relax, repeat 10–15 times as a set, perform 3–4 sets daily to promote pelvic floor muscle function recovery.

Stoma care guidance (for patients with stoma): Conducted 2–3 times during hospitalization (once on postoperative day 3, then once every 2–3 days) and once a month during post-discharge follow-up, 45–60 minutes each time. Professional nurses explain stoma care knowledge to patients and families in detail, including stoma bag selection, replacement methods, skin care. Demonstrate the correct stoma care procedure and allow patients and families to practice until proficient. Regularly organize stoma patient exchange meetings, invite experienced patients to share stoma care experiences and life experiences, and improve patients’ self-care ability and quality of life.

(4) Social support care

Family support: Conducted once during hospitalization (on postoperative day 4) and once during the first post-discharge follow-up, 60 minutes each time. Provided health education to patients’ family members so that they understand colon cancer, the changes in patients’ psychological state after surgery, and the importance of family support for patient recovery. Guide family members to provide care, attention and encouragement in daily life, create a good family atmosphere, and actively participate in patient’s care, such as assisting with rehabilitation training and supervising diet.

Utilization of social resources: Implemented once during the first post-discharge follow-up. Help patients connect with social resources such as community medical service institutions and cancer rehabilitation organizations to provide more rehabilitation support and information exchange platforms. Encourage patients to participate in social activities and return to normal life to enhance their sense of social belonging and self-confidence. For example, introduce the local cancer rehabilitation club to patients and encourage them to participate in club activities and interact with other cancer patients to jointly cope with disease challenges.

### Clinical Observation Indicators

#### Psychological State Assessment Indicators

Self-Rating Anxiety Scale (SAS): Assess patients before surgery, 1 week after surgery, 1 month after surgery, and 3 months after surgery. The scale contains 20 items, each scored 1–4. The standard score is the integer obtained by multiplying the sum of the scores by 1.25. A standard score <50 indicates no anxiety, 50–59 mild anxiety, 60–69 moderate anxiety, and >70 severe anxiety [21, 22]. In this study, the Cronbach’s α coefficient of the SAS was 0.832, and the content validity index (CVI) was 0.86, indicating good reliability and validity. Compare SAS scores between the two groups at each time point to evaluate the effect of comprehensive nursing care on patients’ anxiety.

Self-Rating Depression Scale (SDS): Assessment performed at the same time points. The scale has 20 items, each scored 1–4. The standard score is the integer obtained by multiplying the total score by 1.25. A standard score <53 indicates no depression, 53–62 mild depression, 63–72 moderate depression, and >73 severe depression [23, 24]. In this study, the Cronbach’s α coefficient of the SDS scale was 0.815, and the CVI was 0.84, showing good reliability and validity. Analyse changes in patients’ depression levels based on extracted scores to assess the effect of nursing care.

#### Rehabilitation-Related Indicators

Gastrointestinal function recovery time: Record the time to first anal exhaust and first defecation after surgery. Anal exhaust time reflects the return of gastrointestinal peristalsis, and defecation time further reflects overall recovery of intestinal function. Compare differences between the two groups in these indicators to evaluate the effect of nursing care on prompting gastrointestinal function recovery.

Hospitalization duration: The number of days from admission for surgery to discharge, reflecting the patient’s overall postoperative recovery speed. Shorter hospitalization duration usually indicates good recovery and can serve as an important indicator to evaluate the effectiveness of nursing measures. Compare the mean hospitalization duration between the two groups.

Complication rate: Observe and record the occurrence of various postoperative complications in both groups, such as incision infection, pulmonary infection, urinary system infection, anastomotic leakage, lower extremity venous thrombosis. The complication rate was calculated as the number of patients with complications divided by the total number of patients, multiplied by 100%, and was compared between groups.

#### Quality of Life Assessment Indicators

Short-Form 36 (SF-36) Health Survey: Assess before and 3 months after surgery. The scale covers 8 domains: physical functioning, role limitations due to physical and emotional problems, bodily pain, general health perceptions, vitality, social functioning, and mental health, with 36 items in total. Each domain score ranges from 0 to 100, with higher scores indicating better quality of life [25]. In this study, the Cronbach’s α coefficient of the SF-36 scale was 0.895, and the CVI was 0.88, indicating good reliability and validity.

#### Sleep Quality

PSQI: Assess before and 3 months after surgery. The scale assesses sleep quality over the past month, including 7 components: subjective sleep quality, sleep latency, sleep duration, sleep efficiency, sleep disturbances, use of hypnotic medications, and daytime dysfunction. Each component is scored 0–3, and the sum of component scores is the PSQI total score(range 0–21), with higher score indicating worse sleep quality. The global cutoff for this scale is a total score >5 [26, 27]. After domestic validation, a score >7 was determine as a more suitable cutoff for Chinese people [18]. In this study, the Cronbach’s α coefficient of the PSQI scale was 0.827, and the CVI was 0.85, showing good reliability and validity. Compare PSQI total scores and component scores between the two groups at different time points to analyse the effect of comprehensive nursing care on patients’ sleep quality.

### Statistical Analysis

SPSS25.0 software (IBM Corp., Armonk, NY, USA) was used for data processing and analysis. All statistical tests were two-sided. After Shapiro–Wilk test, all continuous variables followed a normal distribution and are presented as mean ± standard deviation. Independent-samples *t*-tests were used for two-group comparisons. For categorical variables, $\chi^{2}$ test or Fisher’s exact test was used for between-group comparisons, as appropriate. For repeated measurement indicators (e.g., SAS, SDS, and SF-36), linear mixed models (LMM) were used to analyse the main effects of group, time, and the group × time interaction. For PSQI with only two time points (baseline and 3 months postoperatively), paired *t*-test was used for intra-group comparison and independent-samples *t*-test for inter-group comparison.

For intragroup differences in repeated measures (comparisons of different time points to pre-care baseline), Dunnett’s test was used. For inter-group differences at the same time point, independent-samples *t*-test was used. Effect sizes (Cohen’s d for pairwise comparisons, partial $\eta^{2}$ for LMM) and 95% confidence interval (CI) were reported for key outcomes. A *p* < 0.05 was considered statistically significant.

## Results

### General Information

After PSM, all covariates were well balanced (SMD < 0.1) between the two groups (*p* > 0.05), indicating good comparability (Table 1).

**Table 1.** **General clinical data of patients in two groups**.

Characters		Original cohort				Post-PSM cohort (1:1 matched)				
		Routine nursing group (57)	Comprehensive nursing group (64)	*t/χ^2^*	*p*	Routine nursing group (52)	Comprehensive nursing group (52)	*t/χ^2^*	*p*	SMD
Age (years)		55.26 ± 7.16	56.53 ± 7.63	0.939	0.350	55.15 ± 6.42	55.79 ± 6.18	0.456	0.649	0.082
Sex				0.014	0.906			0.038	0.844	0.056
	Male	30 (52.63%)	33 (51.56%)			27 (51.92%)	26 (50.00%)			
	Female	27 (47.37%)	31 (48.44%)			25 (48.08%)	26 (50.00%)			
BMI (kg/m^2^)		22.61 ± 2.49	23.06 ± 2.12	1.069	0.287	23.18 ± 2.41	23.39 ± 2.30	0.412	0.681	0.075
Education level, n (%)				0.048	0.997			0.177	0.981	0.061
	Primary school and below	10 (17.54%)	12 (18.75%)			9 (17.31%)	8 (15.38%)			
	Junior high school	22 (38.60%)	25 (39.06%)			20 (38.46%)	21 (40.38%)			
	Senior high school/technical secondary school	15 (26.32%)	16 (25.00%)			14 (26.92%)	15 (28.85%)			
	College and above	10 (17.54%)	11 (17.19%)			9 (17.31%)	8 (15.38%)			
Lifestyle (smoking/drinking), n (%)				0.002	0.969			0.046	0.830	0.049
	Yes	18 (31.58%)	20 (31.25%)			16 (30.77%)	15 (28.85%)			
	No	39 (68.42%)	44 (68.75%)			36 (69.23%)	37 (71.15%)			
Tumour location, n (%)				0.004	0.952			0.044	0.833	0.058
	Right colon	19 (33.33%)	21 (32.81%)			17 (32.69%)	16 (30.77%)			
	Left colon (including sigmoid colon)	38 (66.67%)	43 (67.19%)			35 (67.31%)	36 (69.23%)			
Colon cancer TNM staging (n (%))				0.129	0.988			0.182	0.980	0.065
	Stage I	12 (21.05%)	15 (23.44%)			11 (21.15%)	10 (19.23%)			
	Stage II	20 (35.09%)	22 (34.38%)			18 (34.62%)	19 (36.54%)			
	Stage III	18 (31.58%)	20 (31.25%)			17 (32.69%)	16 (30.77%)			
	Stage IV	7 (12.28%)	7 (10.94%)			6 (11.54%)	7 (13.46%)			
Surgical method (n (%))				0.003	0.956			0.000	1.000	0.000
	Open surgery	14 (24.560%)	16 (25.00%)			13 (25.00%)	13 (25.00%)			
	Laparoscopic surgery	43 (75.44%)	48 (75.00%)			39 (75.00%)	39 (75.00%)			

BMI, body mass index; PSM, propensity score matching; TNM, tumour node metastasis; SMD, standardized mean difference.

### Psychological State Assessment

No statistically significant difference was observed in SAS and SDS scores between the two groups before surgery (*p* > 0.05). LMM analysis showed significant main effect of time, groups, and the group × time interaction(all *p* < 0.001). In both groups, SAS and SDS scores at all postoperative time were significantly lower than preoperative scores (*p* < 0.05, Dunnett’s test). At each postoperative time point, score in the comprehensive nursing group were significantly lower than those in the routine nursing group (*p* < 0.05, independent-samples *t*-test), with a more obvious decrease (Tables 2 and 3).

**Table 2.** **SAS scores of patients in two groups at different time points (Mean ± SD, points)**.

Group (n)	SAS				Group main effect (SAS), F/*p*/Partial $\eta^{2}$	Time main effect (SAS), F/*p*/Partial $\eta^{2}$	Group × Time interaction, F/*p*/Partial $\eta^{2}$
	Before surgery	1 week after surgery	1 month after surgery	3 months after surgery			
Routine nursing group (52)	51.36 ± 4.75	48.32 ± 4.72^*^	46.83 ± 4.76^*^	45.36 ± 4.86^*^	105.340/<0.001/0.476	102.480/<0.001/0.674	25.764/<0.001/0.395
Comprehensive nursing group (52)	50.82 ± 5.11	44.34 ± 4.52^*#^	42.26 ± 4.44^*#^	40.13 ± 4.64^*#^			

^*^p < 0.05 vs before surgery (Dunnett’s test); ^#^*p* < 0.05 vs routine nursing group at the same time point (independent-samples *t*-test). SAS, Self-Rating Anxiety Scale.

**Table 3.** **SDS scores of patients in two groups at different time points (Mean ± SD, points)**.

Group (n)	SDS				1.8cmGroup main effect (SDS), F/*p*/Partial $\eta^{2}$	Time main effect (SDS), F/*p*/Partial $\eta^{2}$	Group × Time interaction, F/*p*/Partial $\eta^{2}$
	Before surgery	1 week after surgery	1 month after surgery	3 months after surgery			
Routine nursing group (52)	51.84 ± 6.18	49.85 ± 5.84^*^	49.18 ± 5.14^*^	48.63 ± 4.88^*^	98.523/<0.001/0.442	95.314/<0.001/0.672	28.420/<0.001/0.428
Comprehensive nursing group (52)	52.46 ± 5.64	46.32 ± 5.34^*#^	44.84 ± 5.26^*#^	43.48 ± 5.14^*#^			

^*^p < 0.05 vs before surgery (Dunnett’s test); ^#^*p* < 0.05 vs routine nursing group at the same time point (independent-samples *t*-test). SDS, Self-Rating Depression Scale.

### Rehabilitation-Related Indicators

The comprehensive nursing group had significantly shorter first anal exhaust time (36.09 ± 4.37 hours vs. 48.84 ± 5.54 hours), first defecation time (2.75 ± 0.78 days vs. 3.16 ± 0.73 days) and hospitalization duration (10.05 ± 1.47 days vs. 11.84 ± 1.32 days) than the routine nursing group (All *p* < 0.05, Table 4).

**Table 4.** **Gastrointestinal function recovery time and hospital stay of two groups (Mean ± SD)**.

Group (n)	First exhaust time (hours)	Time of the first defecation (days)	Hospital stay (days)
Routine nursing group (52)	48.84 ± 5.54	3.16 ± 0.73	11.84 ± 1.32
Comprehensive nursing group (52)	36.09 ± 4.37	2.75 ± 0.78	10.05 ± 1.47
*t*	–14.114	–2.972	–10.651
*p*	<0.001	0.004	<0.001

The overall complication rate was 13.46% (7 cases) in the routine nursing group and 3.85% (2 cases) in the comprehensive nursing group. Fisher’s exact test showed no statistically significant difference between groups (*p* = 0.080, Table 5).

**Table 5.** **Postoperative complications of patients in two groups [n (%)]**.

Group (n)	Incision infection	Lung infection	Urinary system infection	Anastomotic fistula	Lower extremity venous thrombosis	Overall incidence
Routine nursing group (52)	1 (1.92)	0 (0.00)	2 (3.85)	2 (3.85)	2 (3.85)	7 (13.46)
Comprehensive nursing group (52)	0 (0.00)	1 (1.92)	0 (0.00)	1 (1.92)	0 (0.00)	2 (3.85)
Fisher’s exact test *p*						0.080

### Quality of Life Assessment

No statistically significant difference was observed in any SF-36 domain score between the two groups before surgery (*p* > 0.05). LMM analysis showed significant main effects of time, group, and group × time interaction (all *p* < 0.001). In both groups, scores in all domains at all postoperative time points were significantly higher than preoperative scores (*p* < 0.05). At each time point, the comprehensive nursing group had significantly higher scores than the routine nursing group (*p* < 0.05), with greater improvements (Table 6).

**Table 6.** **SF-36 scores of patients in two groups at different time points (Mean ± SD, points)**.

Item	Before surgery		1 week after surgery		1 month after surgery		3 months after surgery		Group main effect, F/*p* /Partial $\eta^{2}$	Time main effect, F/*p*/ Partial $\eta^{2}$	Group × Time interaction, F/*p*/ Partial $\eta^{2}$
	Routine nursing group (52)	Comprehensive nursing group (52)	Routine nursing group (52)	Comprehensive nursing group (52)	Routine nursing group (52)	Comprehensive nursing group (52)	Routine nursing group (52)	Comprehensive nursing group (52)			
Physiological function	61.58 ± 6.48	61.15 ± 6.63	65.24 ± 6.52^*^	72.18 ± 7.04^*#^	68.37 ± 6.70^*^	76.86 ± 7.24^*#^	70.92 ± 6.86^*^	81.28 ± 7.55^*#^	34.571/<0.001/0.424	98.655/<0.001/0.676	42.145/<0.001/0.483
Role physical	60.64 ± 6.17	61.12 ± 6.58	64.34 ± 6.23^*^	71.08 ± 7.16^*#^	67.56 ± 6.44^*^	75.32 ± 7.34^*#^	70.94 ± 6.55^*^	81.23 ± 7.56^*#^	27.253/<0.001/0.312	89.322/<0.001/0.642	28.634/<0.001/0.426
Somatic pain	61.63 ± 6.56	61.15 ± 6.52	65.14 ± 6.42^*^	72.33 ± 7.15^*#^	68.22 ± 6.54^*^	77.16 ± 7.27^*#^	70.95 ± 6.39^*^	81.03 ± 7.37^*#^	31.292/<0.001/0.295	92.562/<0.001/0.652	25.322/<0.001/0.386
General health	59.77 ± 6.65	60.45 ± 6.12	63.25 ± 6.71^*^	70.15 ± 6.98^*#^	66.58 ± 6.62^*^	75.89 ± 7.15^*#^	71.02 ± 6.73^*^	81.16 ± 7.46^*#^	23.574/0.001/0.178	85.782/<0.001/0.634	20.150/0.001/0.312
Vitality	61.34 ± 5.86	60.73 ± 6.53	64.84 ± 5.97^*^	71.51 ± 6.84^*#^	67.94 ± 6.12^*^	76.36 ± 7.04^*#^	70.71 ± 6.45^*^	80.83 ± 7.35^*#^	34.312/<0.001/0.244	90.665/<0.001/0.634	32.568/<0.001/0.446
Social function	61.47 ± 6.32	60.75 ± 6.43	65.38 ± 6.22^*^	72.06 ± 6.94^*#^	68.56 ± 6.32^*^	77.03 ± 7.17^*#^	70.92 ± 6.43^*^	81.23 ± 7.42^*#^	35.281/<0.001/0.278	96.332/<0.001/0.672	40.889/<0.001/0.472
Emotional function	60.52 ± 6.28	61.12 ± 6.21	64.22 ± 6.44^*^	71.85 ± 6.84^*#^	67.83 ± 6.52^*^	76.94 ± 7.03^*#^	70.87 ± 6.65^*^	81.26 ± 7.44^*#^	56.145/<0.001/0.651	105.223/<0.001/0.712	51.366/<0.001/0.564
Mental health	61.15 ± 6.46	60.64 ± 6.82	68.26 ± 6.52^*^	78.38 ± 7.24^*#^	72.14 ± 6.88^*^	82.03 ± 7.85^*#^	76.36 ± 7.15^*^	85.94 ± 8.03^*#^	67.646/<0.001/0.574	112.564/<0.001/0.723	65.892/<0.001/0.633

^*^p < 0.05 vs baseline (Dunnett’s test); ^#^*p* < 0.05 vs routine nursing group at the same time point (independent-samples *t*-test); Partial $\eta^{2}$: effect size of group × time interaction in LMM.

### Sleep Quality Assessment

Before surgery, no statistically significant difference was observed in PSQI total or component scores between the two groups (*p* > 0.05). Paired *t*-test showed that PSQI scores at 3 months postoperatively were significantly lower than baseline in both groups (*p* < 0.05). Independent-samples *t*-test showed that the comprehensive nursing group had significantly lower PSQI total score and several component scores than the routine nursing group at 3 months postoperatively (*p* < 0.05, Table 7).

**Table 7.** **PSQI scores of patients in two groups (Mean ± SD, points)**.

Item	Before surgery (baseline)		*t*	*p*	3 months postoperatively		*t*	*p*
	Routine nursing group (52)	Comprehensive nursing group (52)			Routine nursing group (52)	Comprehensive nursing group (52)		
Sleep quality	1.54 ± 0.50	1.55 ± 0.50	0.033	0.974	1.28 ± 0.45^*^	1.08 ± 0.27^*^	–3.022	0.003
Sleep latency	1.61 ± 0.49	1.58 ± 0.50	–0.399	0.691	1.00 ± 0.00^*^	1.19 ± 0.39^*^	3.597	<0.001
Sleep efficiency	1.68 ± 0.47	1.66 ± 0.48	–0.324	0.747	1.07 ± 0.26^*^	1.06 ± 0.24^*^	–0.168	0.867
Sleep time	1.67 ± 0.48	1.69 ± 0.47	0.243	0.809	1.04 ± 0.19^*^	1.03 ± 0.18^*^	–0.117	0.907
Hypnotic drugs	1.46 ± 0.50	1.52 ± 0.50	0.649	0.517	1.16 ± 0.41^*^	1.02 ± 0.22^*^	–2.404	0.018
Sleep disturbances	1.51 ± 0.50	1.48 ± 0.50	–0.266	0.791	1.05 ± 0.23^*^	1.05 ± 0.21^*^	–0.144	0.885
Daytime dysfunction	1.54 ± 0.50	1.56 ± 0.50	0.204	0.839	0.91 ± 0.34^*^	1.09 ± 0.29^*^	3.318	0.002
Total score	11.16 ± 1.42	11.22 ± 1.55	0.224	0.823	8.14 ± 0.81^*^	6.27 ± 0.65^*^	–14.107	<0.001

^*^p < 0.05 vs baseline (paired *t*-test).

## Discussion

This study identified a significant positive correlation between comprehensive nursing care and improved psychological state, rehabilitation outcomes, quality of life, and sleep quality in patients with colon cancer who developed postoperative sleep disorders, which is consistent with a recent relevant study [28]. The core value of comprehensive nursing care lies in its multidimensional integrated design, which overcomes the limitations of traditional single physiological nursing and provides systematic support for patients’ physical and mental recovery.

Regarding psychological state improvement, personalized psychological counselling plays a key role. By providing targeted disease education and cognitive correction based on patients’ psychological characteristics, it effectively alleviates anxiety and depression—consistent with the cognitive–behavioural therapy theory, which emphasizes emotional regulation through cognitive adjustment [29]. Such psychological support helps patients establish a positive rehabilitation attitude, laying a foundation for improvements in other outcomes.

Regarding rehabilitation-related indicators, comprehensive nursing care was positively correlated between faster gastrointestinal function recovery and shorter hospitalization duration. Early activity guidance stimulates gastrointestinal reflexes, promotes intestinal peristalsis recovery and reduces the risk of complications such as intestinal adhesion. For the significant difference in hospitalization duration, PSM sensitivity analysis (52 matched pairs) further verified that this correlation is not affected by residual confounding factors such as disease severity, and is closely related to the nursing mode itself rather than pathway or temporal effects. Although the complication rate in the comprehensive nursing group (3.85%) was lower than in the routine nursing group (13.46%), the difference was not statistically significant (*p* = 0.080), which may be due to the limited sample size. Future studies with larger samples are needed to confirm the potential protective effect of comprehensive nursing care on reducing complications.

Regarding quality of life, the comprehensive improvement in SF-36 scores in the comprehensive nursing group reflects the synergistic effect of multimodule care: pain management relieves somatic discomfort and enhances patients’ participation in daily activities; psychological nursing and social support improve psychosocial function—this fully aligns with the modern ‘biological–psychological–social’ medical model [30].

For sleep quality, analysis of covariance analysis (adjusted for baseline) showed that comprehensive nursing care is significantly correlated with improved sleep outcomes. This may result from the synergistic effect of multiple measures: psychological counselling corrects pain catastrophizing cognition, reducing sleep interruption caused by pain-related rumination; pain management inhibits hypothalamic–pituitary–adrenal axis activation and reduces cortisol release that disrupts sleep [31]; early rehabilitation regulates circadian rhythm, and social support alleviates loneliness [32]. Compared with studies using home exercise combined with cognitive–behavioural therapy [33], the improvement in sleep quality in this study is relatively moderate, suggesting that personalized sleep care programs tailored to individual differences may further optimize outcomes.

It should be noted that subjective indicators such as SAS, SDS, and SF-36 may be affected by social desirability bias. Although unified assessor training was conducted to standardize the process, future studies can supplement with objective indicators (e.g., salivary cortisol, polysomnography) to improve result reliability.

This study has several limitations: firstly, the retrospective non-randomized design may introduce selection bias and confounding; secondly, single-centre data limit the generalizability of the results; thirdly, the relatively small sample size may affect detection of subtle care effects; fourthly, the short follow-up time does not allow full evaluation of long-term outcomes such as recurrence rate. Future research should adopt multicentre randomized designs, expand sample size, and extend follow-up time to further verify the long-term correlation between comprehensive nursing care and patient outcomes.

## Conclusions

This study indicates a significant positive correlation between comprehensive nursing care and improved health outcomes in patients with colon cancer who developed postoperative sleep disorders: it is associated with alleviated anxiety and depression, faster gastrointestinal function recovery, shorter hospitalization duration, and better quality of life and sleep quality. Although the difference in complication rate between the two groups was not statistically significant, the comprehensive nursing group had fewer complications, and patients showed high acceptance of this nursing model. Limited by single-centre, sample size and follow-up duration, this study has certain limitations. Future research should adopt a multicentre design, expand the sample size, extend follow-up, incorporate more influencing factors, and optimize evaluation indicators to further verify the value of comprehensive nursing care, and provide high-quality personalized nursing services for postoperative patients with colon cancer to advance clinical nursing practice.

## Availability of Data and Materials

The datasets used and/or analyzed during the current study are available from the corresponding author on reasonable request.
